# Antibacterial and Antioxidant Properties of the Methanolic Extract of the Stem Bark of *Pteleopsis hylodendron* (Combretaceae)

**DOI:** 10.1155/2011/218750

**Published:** 2011-03-03

**Authors:** Aristide Laurel Mokale Kognou, Rosalie Annie Ngono Ngane, Jules Roger Kuiate, Martin Luther Koanga Mogtomo, Alembert Tchinda Tiabou, Raymond Simplice Mouokeu, Lucie Biyiti, Paul Henri Amvam Zollo

**Affiliations:** ^1^Laboratory of Microbiology and Antimicrobial Substances, University of Dschang, P.O. Box 67 Dschang, Cameroon; ^2^Department of Biochemistry, University of Douala, P.O. Box 24157 Douala, Cameroon; ^3^Laboratory of Phytochemistry, Institute of Medical Research and Medicinal Plants Study, Ministry of Scientific Research and Innovation, P.O. Box 6163, Yaoundé, Cameroon; ^4^Department of Biochemistry, University of Yaoundé I, P.O. Box 812 Yaoundé, Cameroon

## Abstract

*Pteleopsis hylodendron* (Combretaceae) is used in Cameroon and West Africa folk medicine for the treatment of various microbial infections (measles, chickenpox, and sexually transmitted diseases). The antibacterial properties of the methanolic extract and fractions from stem bark of *Pteleopsis hylodendron* were tested against three Gram-positive bacteria and eight Gram-negative bacteria using Agar-well diffusion and Broth microdilution methods. Antioxidant activities of the crude extract and fractions were investigated by DPPH radical scavenging activity and *β*-carotene-linoleic acid assays. The methanolic extract and some fractions exhibited antibacterial activities that varied between the bacterial species (ID = 0.00–25.00 mm; MIC = 781–12500 *μ*g/mL and 0.24–1000 *μ*g/mL). The activity of the crude extract is, however, very weak compared to the reference antibiotics (MIC = 0.125–128 *μ*g/mL). Two fractions (F_E_ and F_F_) showed significant activity (MIC = 0.97 *μ*g/mL) while *S. aureus* ATCC 25922 was almost resistant to all the tested fractions. In addition, the crude extract and some fractions showed good antioxidant potential with inhibition values ranging from 17.53 to 98.79%. These results provide promising baseline information for the potential use of this plant as well as some of the fractions in the treatment of infectious diseases and oxidative stress.

## 1. Introduction

Since the successive introduction of various antibiotics into therapeutics, the sensitivity of pathogenic microorganisms changed a lot so that the proportion of antibiotically resistant strains is currently important [1], what involves an increase in seriousness of infectious diseases as gastroenteritis (GE) which are a problem of public health on a worldwide scale but especially in Africa [2]. Diarrhea, its main characteristic is a major cause of morbidity and mortality among children in developing countries. According to the World Health Organization (WHO), there are more than 2 million deaths per year [3]. Moreover, therapy with synthetic antibiotics is not always possible because of their high cost as well as toxicity due to their extended use. To overcome this problem, people in developing countries use preparations obtained from plants following folk tradition for their primary health care because of low cost with little or no undesirable side effects [4]. The plants represent a potential and almost inexhaustible source of new anti-infective compounds [5] and many of them are used to treat GE effectively [6].

*Pteleopsis hylodendron* Mildbr. belongs to the family Combretaceae commonly found in the forest regions of West and Central Africa. The genus *Pteleopsis* is represented in Africa by ten species but only *P. hylodendron* is found in Cameroon [7]. The aqueous decoction of the stem bark of *P. hylodendron* is used to treat measles, chickenpox, sexually transmitted diseases, GE, female sterility, liver and kidney disorders, as well as dropsy [8]. Phytochemical, antimicrobial, toxicity and antioxidant works have been previously reported of this plant [9, 10]. In the same logic, we have analysed the stem bark of *Pteleopsis hylodendron* and report here the antibacterial activity on pathogenic bacteria of the gastro-intestinal tract and antioxidant activity of the crude extract and fractions.

## 2. Material and Methods

### 2.1. Plant Material

The stem bark of *P. hylodendron* was collected in February 2009 at Mbeyengue I, Centre region of Cameroon. Identification was done at the National Herbarium in Yaoundé, Cameroon, where a voucher specimen (No 1309/SFRK) has been deposited.

### 2.2. Microorganisms

Five bacterial strains and six isolates known to be pathogenic of the gastro-intestinal tract were used in this work. These included three Gram**^+^** bacteria (*Enterococcus faecalis *ATCC 10541, *Staphylococcus aureus *ATCC 25922 and *Staphylococcus aureus*) and eight Gram^−^ bacteria (*Escherichia coli* ATCC 11775,* Escherichia coli*, *Proteus mirabilis*,* Pseudomonas aeruginosa *ATCC 27853,* Salmonella paratyphi A, Salmonella paratyphi B, Salmonella typhi *ATCC 6539,* and Shigella flexeneri*). The bacterial isolates were obtained from Centre Pasteur of Yaoundé, Cameroon, while the reference strains were obtained from American Type Culture Collection (ATCC). The bacterial strains and isolates were grown at 35°C and maintained on nutrient agar. The bacterial cell suspension was prepared at 1.5 × 10^8^ colony forming units per mL (CFU/mL) following the McFarland 0.5 turbidity standard.

### 2.3. Extraction and Fractionation

The air-dried and powdered stem bark of *Pteleopsis hylodendron* (2.5 kg) was extracted with MeOH (8 L, 72 h) at room temperature to obtain a crude extract (590 g) after evaporation under vacuum. A portion of this extract (100 g) was subjected to silica gel column chromatography (Ø8 cm × L30 cm) eluted successively with pure hexane, hexane-EtOAc (90 : 10–30 : 70), pure EtOAc, EtOAc-MeOH (95 : 5–80 : 20) and pure MeOH. Forty-six fractions of 500 mL each were collected and combined based on their TLC profile into ten major fractions A–J (A: 2-3, B: 4–6, C: 7–13, D: 14–16, E: 17–21, F: 22–28, G: 29–35, H: 36-37, I: 38–44, J: 45–46).

### 2.4. Antibacterial Assays

#### 2.4.1. Agar-Well Diffusion Method

Diameters of inhibition zones (ID) were determined using Mueller Hinton Agar (MHA) by the well diffusion method [11]. The bacterial suspension (100 *μ*L) was homogeneously seeded onto Petri dishes containing sterile molten MHA (20 mL). The sterile 6 mm diameter wells were impregnated (50 *μ*L) with different concentrations of plant extract (10, 5, and 2.5 mg–200, 100, and 50 *μ*g/mL). The dishes were kept for 1 h at room temperature for the diffusion of the extract. Subsequently, dishes were incubated at 35°C for 24 h. Antibiotics (Amoxicillin, Ciprofloxacin and Gentamicin) were used as positive control (10 *μ*g–200 *μ*g/mL) and 10% aqueous DMSO was used as negative control. Results were evaluated by measuring the inhibition zones around each well. The assay was done in triplicate and the mean diameters recorded as inhibition zones. We considered that an extract is active when ID was up to 20 mm, and then the strain is known as sensitive; moderately active when ID was between 10 and 20 mm, and then the strain is known as moderate; little or not active when ID was between 0 and 10 mm, and then the strain is known as little sensitive or resistant [12, 13].

#### 2.4.2. Broth Microdilution Method

Minimum inhibitory concentrations (MICs) were determined using Mueller Hinton Broth (MHB) by microdilution method [14]. A two-fold serial dilution of the crude extract (12.50–0.024 mg/mL) and fractions (1000–1.953 *μ*g/mL and 500–0.242 *μ*g/mL). A negative control (10%, v/v aqueous DMSO, medium and inoculum) and positive control (10%, v/v aqueous DMSO, medium, inoculum and water-soluble antibiotics) were included. Each well of 96-well sterile microtitre plate received 100 *μ*L of MHB, 100 *μ*L of test substances and 100 *μ*L of the bacterial inoculum (1.5 × 10^8^ CFU/mL). The plates were covered and incubated at 35°C for 24 h. As an indicator of bacterial growth, 50 *μ*L *p*-iodonitrotetrazolium violet (INT) dissolved in water was added to the wells and incubated at 35°C for 30 min. MIC values are recorded as the lowest concentration of the substance that completely inhibited bacterial growth that is, the solution in the well remained clear after incubation with INT. Minimum bactericidal concentrations (MBCs) were determined by plating 10 *μ*L from each negative well and from the positive growth control on Mueller Hinton Agar. MBCs were defined as the lowest concentration yielding negative subcultures. The experiments were performed in triplicate. Amoxicillin, ciprofloxacin and gentamicin at the concentration ranging between 128 and 0.062 *μ*g/mL served as positive control.

### 2.5. Antioxidant Assay

#### 2.5.1. DPPH Assay

The free radical scavenging activity of the extract and fractions on the stable radical DPPH were estimated by the method of Mensor et al. [15]. 1.5 mL of a methanol solution of sample test at different concentrations (10, 50, 100, 500, and 1000 *μ*g/mL) was mixed with a 0.3 mM DPPH methanol solution and kept for 30 min at room temperature. The decrease in the solution absorbance, due to proton donating of substances was measured at 517 nm. L-Ascorbic acid was used as positive control. The percentage of DPPH radical scavenging activity was calculated using the following formula:

(1) $$\begin{array}{cc} & \text{DPPH}\;\text{radical}\;\text{scavenging}\;\text{activity}\;\left(\text{\%}\right)\\ & \;\;=\;\left[\frac{\left({\text{A}}_{\text{control}}-{\text{A}}_{\text{sample}\;\;\text{test}}\right)}{{\text{A}}_{\text{control}}}\right]\;\times 100. \end{array}\;$$

#### 2.5.2. *β*-Carotene-Linoleic Acid Assay

In this assay antioxidant capacity is determined by measuring the inhibition of the volatile organic compounds and the conjugated diene hydroperoxides arising from linoleic acid oxidation [16]. A stock solution of *β*-carotene-linoleic acid mixture was prepared as follows: 1.5 mg *β*-carotene was dissolved in 3 mL of chloroform, and 75 *μ*L linoleic acid and 600 mg tween 40 were added. Chloroform was completely evaporated using a vacuum evaporator. Then 150 mL distilled water saturated with oxygen was added with a vigorous shaking. 1340 *μ*L of this reaction mixture was dispersed to test tubes and 160 *μ*L extract (20 mg/mL) were added, and emulsion system was incubated at 55°C for 105 min. Same procedure was repeated with L-Ascorbic acid used as a standard control and a blank. After this incubation period absorbance of the mixtures were measured at 492 nm. Antioxidative capacity of the extract was compared with those of ascorbic acid and blank.

### 2.6. Phytochemical Screening

Chemical tests were carried out on the methanolic extract and fractions using standard procedures to identify the constituents (alkaloids, anthocyanins, anthraquinones, coumarins, flavonoids, glycosides, phenols, polyphenols, saponins, tannins, triterpenes, and sterols) as described by Brunetton [17].

### 2.7. Statistical Analysis

Data were expressed as mean ± standard deviation. Statistical analysis was carried out using the Waller-Duncan's test. The 12.0 SPSS Windows software was used for this analysis. Differences were considered significant at *P* < .05.

## 3. Results

### 3.1. Extraction and Fractionation

The % yield of methanolic extract of *P. hylodendron* was 15.96%. F_I_ (43.08%) and F_J_ (24.54%) were the most abundant.

### 3.2. Phytochemical Screening

Phytochemical screening revealed the presence of medicinally active constituents. The differences in the composition between crude extract and fractions and between fractions were noted. Except F_A_, all other substances contained at least one chemical group. Alkaloids, anthocyanins, anthraquinones, flavonoids, glycosides, phenols, polyphenols, saponins, and tannins were present in crude extract while coumarins, sterols, and triterpenes were absent. F_B_, F_C_, and F_D_ had a similar chemical composition (alkaloids). It is the same for F_G_ and F_H_ (alkaloids, anthocyanins, anthraquinones, flavonoids, phenols, polyphenols, and tannins); F_I_ and F_J_ (flavonoids, glycosides, phenols, polyphenols, saponins, and tannins).

### 3.3. Antibacterial Activity

The results of the antibacterial activity by the Agar-well diffusion method are presented in Table 1. At the three concentrations of the methanolic extract tested, ID ranged from 0.00 to 25.00 mm for all the bacteria 15.00–25.00 mm for the isolates 0.00–22.00 mm for the Gram^−^, and 10.87–25.00 mm for the Gram^+^. *S. aureus* was the most sensitive (ID = 20.00–25.00 mm) while *S. aureus *ATCC 25922 and *E. coli* ATCC 11775 were the least sensitive (ID = 11.00–15.00 mm and 10.00–14.75 mm resp.). No activity was recorded at 2.5 mg against *P. aeruginosa *ATCC 27853. However these values are weak compared with those of the reference antibiotics (ID = 12–40 mm).

In view of the results obtained by diffusion method, MIC and MBC values of the crude extract and fractions were established and the results are shown in Tables 2 and 3.

All the bacteria tested were inhibited by the methanolic extract (Table 2) with MIC ranging from 781–12500 *μ*g/mL for all the bacteria, isolates, and Gram^−^; 781–3125 *μ*g/mL for the strains and Gram^+^. *S. paratyphi *A was the least sensitive (MIC = 12500 *μ*g/mL). *P. aeruginosa *ATCC 27853, *P. mirabilis* and *S. aureus* were the most sensitive (MIC = 781 *μ*g/mL). The important activity on *S. aureus* confirms the best activity obtained in solid medium; which revealed this germ as one of the most susceptible. Antibiotics exerted a higher inhibitory effect on bacterial (MIC = 0.125–128 *μ*g/mL) than the methanolic extract.

The fractionation of the methanolic extract showed an inactivity of F_A_, F_B_, F_C_ and F_D_ on all the bacteria tested (Table 3). On the contrary, F_E_ and F_F_ saw their activity increasing significantly. Indeed, on five of the eleven bacteria (*E. coli*, *P. mirabilis*, *S. paratyphi B*,* E. faecalis *ATCC 10541, *S. aureus*), MIC was 0.97 *μ*g/mL, making the substances more active than reference antibiotics. F_G_ had a fairly good activity (MIC = 0.24 *μ*g/mL) on some bacteria (*P. aeruginosa *ATCC 27853,* E. faecalis *ATCC 10541, *S. aureus*) whereas F_H_, F_I_ and F_J_ were slightly active (MIC = 125–1000 *μ*g/mL). *S. aureus *ATCC 25922 was almost resistant to all the fractions (MIC > 1000 *μ*g/mL). The MBC/MIC ratio activity for all the bacteria tested varied between one (1) and eight (8) for the crude extract and between one (1) and twenty (20) for the fractions. According to Marmonier [18], plant extract and fractions exerted two types of activities: a bacteriostatic (MBC/MIC ≥ 4) and bactericidal activity (MBC/MIC ≤ 4). Methanolic extract and fractions of *P. hylodendron* were bactericidal on at least 63% and 27% of the bacteria respectively.

### 3.4. Antioxidant Activity

The antioxidant activity of the methanolic extract and fractions was assessed by the DPPH and *β*-carotene-linoleic acid assays. The results are presented in Tables 4 and 5. Activity increased in a concentration-dependant manner compared to L-ascorbic acid (positive antioxidant control). At the concentrations of 500 and 1000 *μ*g/mL the methanolic extract, F_E_, F_F_, F_G_, F_H_, F_I_ and F_J_ showed a similar activity to that one of L-ascorbic acid. F_H_  was the most antioxidant fraction (94.05–98.79%) while F_A_  was the least antioxidant (1.86–21.26%).

## 4. Discussion and Conclusion

Phytochemical screening of the methanolic extract and fractions of *P. hylodendron* revealed the presence alkaloids, anthocyanins, anthraquinones, flavonoids, glycosides, phenols, polyphenols, saponins and tannins. Other investigators [19, 20] have reported the presence of these components in the Combretaceae family to which belongs the studied plant. However, Ngounou et al. [21] and Atta-Ur-Rahman et al. [22] working on the stem bark of *P. hylodendron* collected from East region of Cameroon revealed the presence of triterpenes which were absent in our sample. This difference can be attributed to the difference in the geographical region, soil composition, and age of the plant [17].

The antimicrobial activities of *Pteleopsis* species were reported [19, 23]. Generally, the methanolic extract and some fractions of the stem bark of *P. hylodendron* showed variable antibacterial activities dose-dependant on the eleven bacterial strains and isolates tested. These broad spectra of action could be related to their chemical components [24]. Among these compounds, tannins induce an important antimicrobial activity because they have an ability to inactivate microbial adhesions, enzymes, cell envelope transport proteins, and so forth, [25]. Due to their ability to bind to proteins and metals, tannins also inhibit the growth of microorganisms through substrate and metal ion deprivation [26]. However, differences in chemical composition recorded between the crude extract and some fractions may explain their different degree of antimicrobial properties. Also, the amount of the active components in the crude extract may be diluted and fractionation may have increased their concentrations, thus the activities in the fractions [27]. Moreover, the differences in susceptibility may be explained by the differences in cell wall composition and/or genetic content of plasmids that can be easily transferred among strains [28]. MIC values obtained from the extract by micro-dilution method revealed that *S. aureus* is the most sensitive. It was reported [29] that *S. aureus* is one of the most susceptible bacteria to the plant extracts. These values also showed that the Gram^−^ and Gram^+^ bacteria had a comparable susceptibility. This may suggest that the mode of action of the extract was not related to the cell wall composition. * S. aureus* ATCC 25922 which was inhibited completely by the methanolic extract at 3125 *μ*g/mL, was almost resistant to all the fractions. This may suggest that this microbe required high concentrations of the substance tested and synergic effect of chemical compounds as extract. F_A_, F_B_, F_C_, and F_D_ containing only alkaloids did not show any inhibitory effect on the bacteria tested. This may suggest these compounds which also present in the methanolic extract do not have a detectable antibacterial activity. However, alkaloids were reported to possess antibacterial activities [30]. F_E_ and F_F_, most active had a comparable chemical composition that F_G_. Differences in activity between these fractions could be related to the absence of anthocyanins in F_F_ and anthraquinones in F_E_. Generally, it is difficult at the sight of results of the phytochemical screening to attribute the activities recorded to a chemical compounds group. 

As L-ascorbic acid, the methanolic extract and some fractions showed great antioxidant potentials. This particularly high activity could be attributed to the presence of phenolic compounds [31]. The antioxidant activity of phenolic compounds is mainly due to their redox properties, which can play an important role in neutralizing free radicals, quenching singlet and triplet oxygen species, or decomposing peroxides [32]. Numerous studies have suggested flavonoids, anthraquinones, anthocyanins and tannins [33, 34] for antioxidant activity. Previous phytochemical investigations on this plant have reported the presence of ellagic acid derivatives as antioxidant source [22]. 

These results provide promising baseline information for the potential use of this plant as well as some of the fractions in the treatment of GE and oxidative stress. F_E_ and F_F_ by their high antibacterial activity could be the base of development of new antibacterial agents with broad spectra. Their purification and pharmacological and toxicity studies are essential.

## Figures and Tables

**Table 1:** ID (mm) of the methanolic extract of *P. hylodendron. *

Bacteria	Crude extract (mg/mL)			Reference antibiotic (*μ*g/mL)		
	*P. hylodendron*			Amoxicillin	Ciprofloxacin	Gentamicin
	200	100	50		200	
Gram^−^ bacteria						
*E. coli* ATCC 11775	14.75 ± 0.17^f^	12.25 ± 0.17^h^	10.00 ± 0.00^g^	19.00 ± 0.26^d^	28.25 ± 0.17^f^	27.00 ± 0.26^f^
*E. coli *	21.75 ± 0.13^b^	19.00 ± 0.00^c^	16.75 ± 0.17^c^	40.00 ± 0.00^a^	27.75 ± 0.17^f^	26.00 ± 0.00^h^
*P. aeruginosa* ATCC 27853	18.62 ± 0.27^d^	13.75 ± 0.17^f^	0.00 ± 0.00^h^	14.50 ± 0.42^g^	32.50 ± 0.17^d^	30.00 ± 0.00^d^
*P. mirabilis *	20.12 ± 0.12^c^	18.00 ± 0.00^d^	16.00 ± 0.00^d^	16.00 ± 0.00^f^	33.00 ± 0.00^c^	33.25 ± 0.17^a^
*S. flexneri *	21.75 ± 0.17^b^	19.75 ± 0.17^b^	17.50 ± 0.18^b^	20.00 ± 0.00^c^	36.50 ± 0.42^a^	32.00 ± 0.00^b^
*S. paratyphi* A	20.25 ± 0.17^c^	17.00 ± 0.00^e^	15.00 ± 0.00^e^	12.00 ± 0.00^h^	30.00 ± 0.00^e^	28.00 ± 0.00^c^
*S. paratyphi* B	19.75 ± 0.17^c^	17.62 ± 0.12^d^	15.12 ± 0.12^e^	14.25 ± 0.17^g^	35.00 ± 0.00^b^	28.25 ± 0.17^e^
*S. typhi* ATCC 6539	22.00 ± 0.00^b^	20.00 ± 0.00^b^	16.87 ± 0.12^c^	12.00 ± 0.00^h^	32.33 ± 0.25^d^	32.00 ± 0.00^b^
Gram^+^ bacteria						
*E. faecalis* ATCC 10541	16.00 ± 0.00^e^	13.25 ± 0.17^g^	10.87 ± 0.12^f^	18.00 ± 0.00^e^	32.00 ± 0.00^d^	31.00 ± 0.00^c^
*S. aureus* ATCC 25922	15.00 ± 0.00^f^	13.75 ± 0.17^f^	11.00 ± 0.00^f^	20.75 ± 0.17^c^	35.50 ± 0.18^c^	30.25 ± 0.17^cd^
*S. aureus *	25.00 ± 0.00^a^	22.00 ± 0.00^a^	20.00 ± 0.00^a^	35.00 ± 0.00^b^	26.00 ± 0.00^h^	26.75 ± 0.24^fh^

^a,b,c,d,e,f,g,h^In the same column, values carrying different letters in superscript are significantly different at *P* ≤ .05 (Waller Duncan test).

Diameters of inhibition zones (ID).

**Table 2:** MIC and MBC (*μ*g/mL) of the methanolic extract of *P. hylodendron. *

	Crude extract			Reference antibiotics								
Bacteria	*P. hylodendron*			Amoxicillin			Ciprofloxacin			Gentamicin		
	MIC	MBC	MBC/MIC	MIC	MBC	MBC/MIC	MIC	MBC	MBC/MIC	MIC	MBC	MBC/MIC
Gram^−^ bacteria												
*E. coli* ATCC 11775	3125	12500	4	128	—	—	4	4	1	16	128	8
*E. coli *	1562	—	—	1	1	1	8	8	1	1	1	1
*P. aeruginosa* ATCC 27853	781	781	1	128	—	—	1	16	16	8	16	2
*P. mirabilis *	781	6250	8	1	8	8	1	1	1	2	16	8
*S. flexneri *	3125	—	—	32	64	2	0.25	1	4	0.25	0.25	1
*S. paratyphi A *	12500	—	—	—	—	—	0.125	0.5	4	2	2	1
*S. paratyphi B *	1562	6250	4	1	8	8	0.5	2	4	2	16	8
*S. typhi* ATCC 6539	1562	6250	4	—	—	—	0.25	2	8	32	128	4
Gram^+^ bacteria												
*E. faecalis* ATCC 10541	1562	6250	4	1	1	1	4	16	4	1	1	1
*S. aureus* ATCC 25922	3125	6250	2	—	—	—	8	8	1	4	16	4
*S. aureus *	781	1562	2	1	—	—	8	8	1	0.25	0.25	1

—:12500 *μ*g/mL for the extract and >128 *μ*g/mL for the reference antibiotics.

**Table 3:** MIC and MBC (*μ*g/mL) of the fractions from chromatography separation of *P. hylodendron. *

Bacteria	Parameters	Fractions										Reference antibiotics		
		F_A_	F_B_	F_C_	F_D_	F_E_	F_F_	F_G_	F_H_	F_I_	F_J_	Amox	Cipro	Genta
Gram^−^ bacteria														
*E. coli* ATCC 11775	MIC	—	—	—	—	500	—	62.5	500	1000	1000	128	4	16
	MBC	—	—	—	—	—	—	—	—	—	—	—	4	128
	MBC/MIC	—	—	—	—	—	—	—	—	—	—	—	1	8
*E. coli *	MIC	—	—	—	—	0.97	0.97	62.5	500	1000	1000	1	8	1
	MBC	—	—	—	—	—	—	250	—	—	—	1	8	1
	MBC/MIC	—	—	—	—	—	—	4	—	—	—	1	8	1
*P. aeruginosa* ATCC 27853	MIC	—	—	—	—	—	—	0.24	1000	1000	1000	128	1	8
	MBC	—	—	—	—	—	—	250	—	—	—	—	16	16
	MBC/MIC	—	—	—	—	—	—	20	—	—	—	—	16	2
*P. mirabilis *	MIC	—	—	—	—	0.97	0.97	—	250	500	1000	1	1	2
	MBC	—	—	—	—	125	62.5	—	—	—	—	8	1	16
	MBC/MIC	—	—	—	—	14	12	—	—	—	—	8	1	8
*S. flexneri *	MIC	—	—	—	—	—	—	—	250	500	1000	32	0.25	0.25
	MBC	—	—	—	—	—	—	—	—	—	—	64	1	0.25
	MBC/MIC	—	—	—	—	—	—	—	—	—	—	2	4	1
*S. paratyphi A *	MIC	—	—	—	—	—	—	500	1000	1000	—	—	0.125	2
	MBC	—	—	—	—	—	—	—	—	—	—	—	0.5	2
	MBC/MIC	—	—	—	—	—	—	—	—	—	—	—	4	1
*S. paratyphi B *	MIC	—	—	—	—	0.97	0.97	500	500	500	1000	1	0.5	2
	MBC	—	—	—	—	—	—	—	—	—	—	8	2	16
	MBC/MIC	—	—	—	—	—	—	—	—	—	—	8	4	8
*S. typhi* ATCC 6539	MIC	—	—	—	—	—	—	500	1000	—	—	—	0.25	32
	MBC	—	—	—	—	—	—	—	—	—	—	—	2	128
	MBC/MIC	—	—	—	—	—	—	—	—	—	—	—	8	4
Gram^+^ bacteria														
*E. faecalis* ATCC 10541	MIC	—	—	—	—	0.97	0.97	0.24	500	1000	1000	1	4	1
	MBC	—	—	—	—	0.97	250	0.24	—	—	—	1	16	1
	MBC/MIC	—	—	—	—	1	16	1	—	—	—	1	4	1
*S. aureus* ATCC 25922	MIC	—	—	—	—	—	—	—	500	—	—	—	8	4
	MBC	—	—	—	—	—	—	—	—	—	—	—	8	16
	MBC/MIC	—	—	—	—	—	—	—	—	—	—	—	1	4
*S. aureus *	MIC	—	—	—	—	0.97	0.97	0.24	500	125	250	1	8	0.25
	MBC	—	—	—	—	—	—	0.97	—	—	—	—	8	0.25
	MBC/MIC	—	—	—	—	—	—	4	—	—	—	—	1	1

F: fraction; Amox: amoxicillin; Cipro: ciprofloxacin; Genta: gentamicin.

—:>1000 *μ*g/mL for the fractions, and >128 *μ*g/mL for the reference antibiotic.

**Table 4:** Antioxidant potential of the crude extract and fractions of *P. hylodendron *and L- ascorbic acid in DPPH assay.

Substances tested	% inhibition concentration (*μ*g/mL)				
	1000	500	100	50	10
F_A_	21.26 ± 0.24^f^	19.69 ± 0.17^i^	11.83 ± 0.47^ef^	05.28 ± 0.26^j^	1.86 ± 0.17^m^
F_B_	22.40 ± 0.94^f^	18.80 ± 0.10^j^	09.19 ± 0.20^f^	08.83 ± 0.34^i^	04.20 ± 0.17^l^
F_C_	42.58 ± 0.50^e^	28.16 ± 0.29^h^	12.31 ± 0.41^e^	10.87 ± 0.38^h^	08.65 ± 0.00^k^
F_D_	67.98 ± 0.72^d^	52.13 ± 0.10^g^	20.24 ± 0.45^d^	17.83 ± 0.28^g^	10.81 ± 0.37^j^
F_E_	95.73 ± 0.26^bc^	94.95 ± 0.00^d^	94.05 ± 0.14^a^	91.77 ± 0.33^cd^	26.30 ± 0.20^h^
F_F_	95.37 ± 0.10^bc^	94.65 ± 0.10^d^	93.75 ± 0.10^a^	93.33 ± 0.14^ab^	83.42 ± 0.48^d^
F_G_	97.83 ± 0.37^ab^	95.55 ± 0.17^c^	94.65 ± 0.17^a^	92.79 ± 0.29^bc^	91.89 ± 0.28^b^
F_H_	98.79 ± 0.33^ab^	96.21 ± 0.13^b^	94.47 ± 0.26^a^	94.29 ± 0.26^a^	94.05 ± 0.20^a^
F_I_	95.49 ± 0.14^bc^	94.83 ± 0.17^d^	93.93 ± 0.33^a^	92.67 ± 0.38^bc^	70.08 ± 0.64^f^
F_J_	95.85 ± 0.00^bc^	95.13 ± 0.14^cd^	93.57 ± 0.30^a^	93.21 ± 0.17^ab^	74.23 ± 0.14^e^
P_H_	95.13 ± 0.14^bc^	93.87 ± 0.28^e^	93.39 ± 0.10^a^	91.35 ± 0.14^d^	89.42 ± 0.26^c^
ASC	100.00 ± 0.00^a^	100.00 ± 0.00^a^	87.36 ± 0.00^b^	53.23 ± 0.00^e^	29.42 ± 0.00^g^

F: fraction; PH: methanolic extract of *P. hylodendron*; ASC: L-ascorbic acid.

^a,b,c,d,e,f,g,h,i,j,k,l,m^In the same column, values carrying different letters in superscript are significantly different at *P* ≤ .05 (Waller Duncan test).

**Table 5:** Antioxidant potential of the crude extract of *P. hylodendron *and L-ascorbic acid in *β*-carotene-linoleic acid assay.

Substances tested	% inhibition concentration (*μ*g/mL)				
	1000	500	100	50	10
P_H_	38.03 ± 0.25^b^	31.83 ± 0.16^a^	31.80 ± 0.00^a^	30.47 ± 0.08^a^	27.73 ± 0.08^a^
ASC	56.48 ± 0.05^a^	27.73 ± 0.02^b^	20.20 ± 0.00^b^	19.86 ± 0.00^b^	17.81 ± 0.02^b^

P_H_: methanolic extract of *P. hylodendron*; ASC: L-ascorbic acid.

^a,b^In the same column, values carrying different letters in superscript are significantly different at *P* ≤ .05 (Waller Duncan test).
